# Supplementary material for: A Meta-analysis Describing the Effects of the Essential oils Blend Agolin Ruminant on Performance, Rumen Fermentation and Methane Emissions in Dairy Cows
Source: Animals (Basel). 2020 Apr 3;10(4):620. doi: 10.3390/ani10040620 (PMC7222807; doi:10.3390/ani10040620)
Supplement: Supplementary file 1 [file animals-10-00620-s001.pdf]

animals

SUPPLEMENTARY MATERIALS

A Meta-analysis Describing the Effects of the Essential Oils Blend Agolin Ruminant on Performance, Digestion and Methane Emissions in Dairy Cows

Alejandro Belanche, Charles J. Newbold, Diego P. Morgavi, Alex Bach, Beatrice Zweifel and David R. Yañez-Ruiz

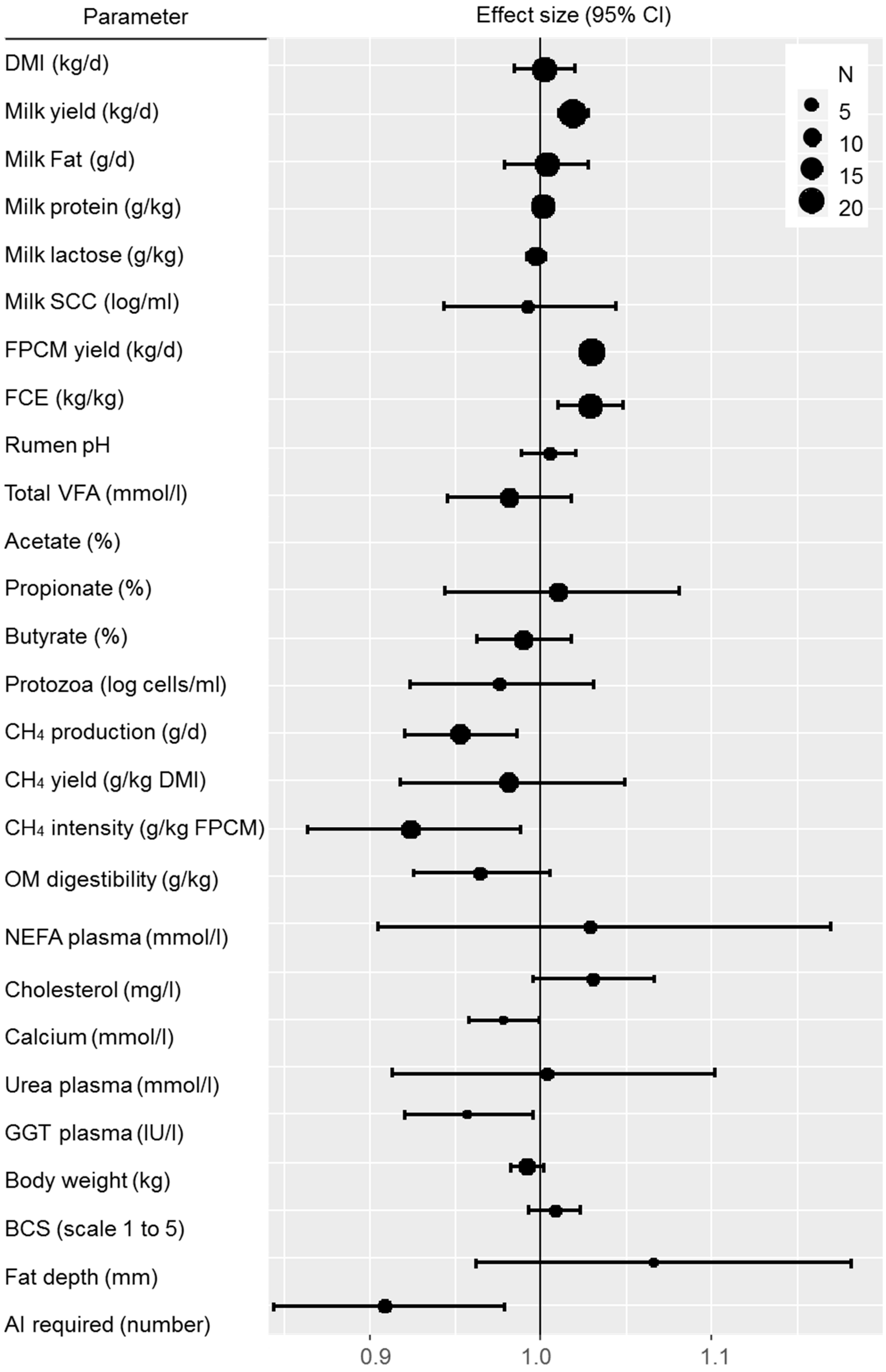

Figure S1. Forest plot describing the overall effects of supplementing an essential oil blend (1 g/d per cow) to dairy cows. Effect size is expressed as response ratio.

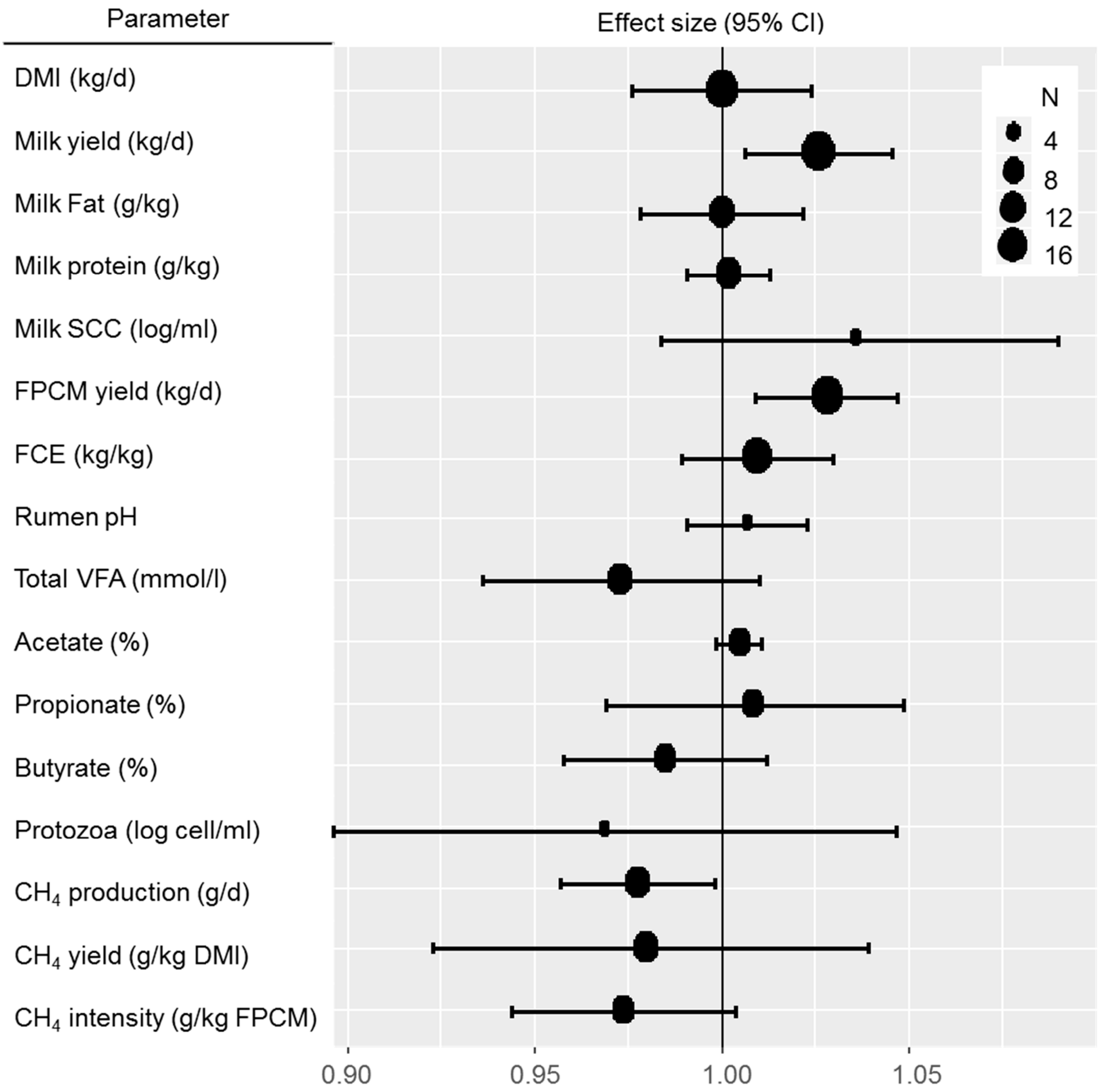

Figure S2. Forest plot describing the short-term effects (<28 days of treatment) of supplementing an essential oil blend (1 g/d) to dairy cows. Effect size is expressed as response ratio.

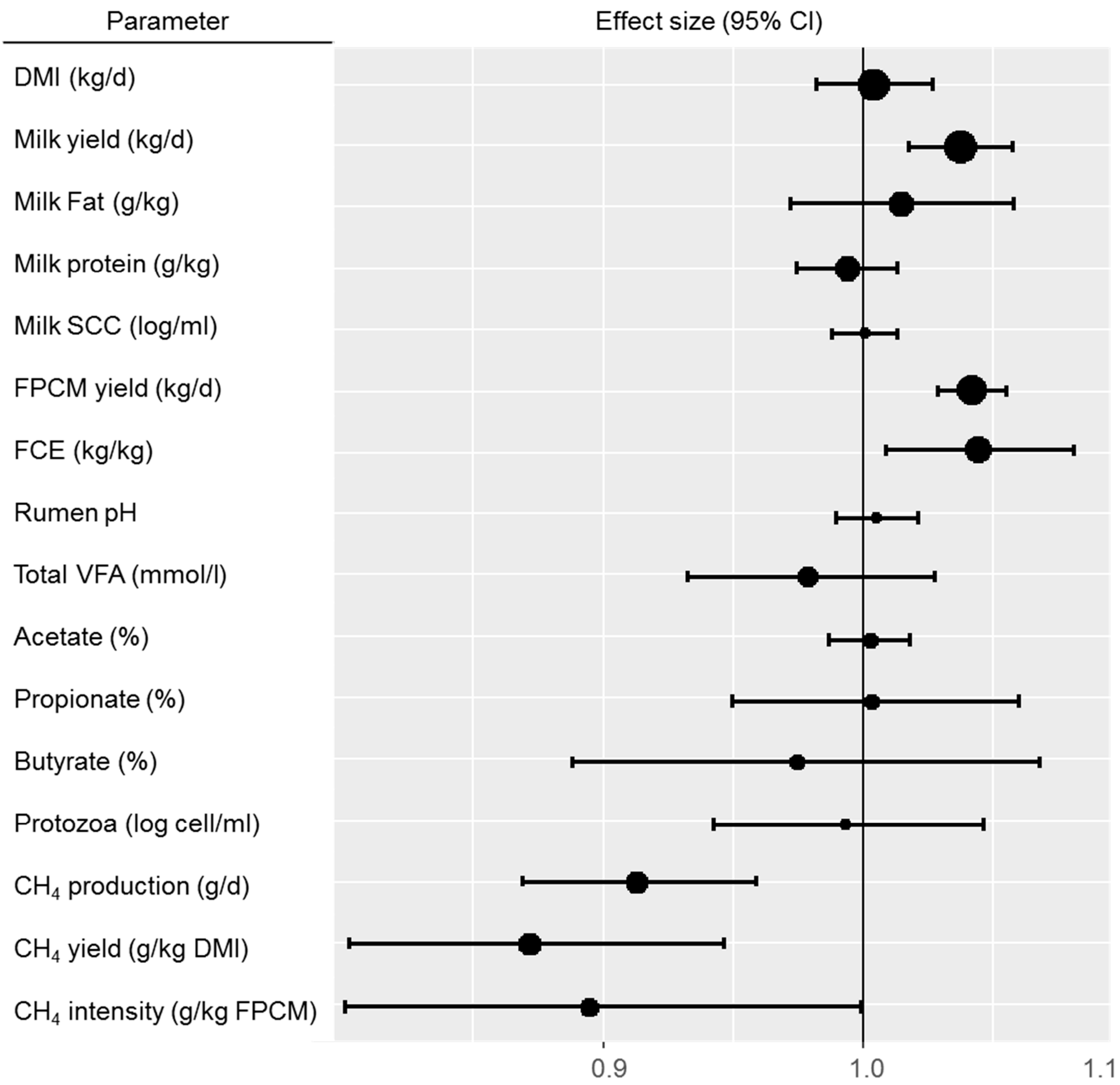

Figure S3. Forest plot describing the long-term effects (≥28 days of treatment) of supplementing an essential oil blend (1 g/d) to dairy cows. Effect size is expressed as response ratio.
